# Extracorporeal membrane oxygenation with single-site dual-lumen in a patient with SARS-CoV-2–associated acute respiratory distress syndrome: a case report

**DOI:** 10.3389/fmed.2026.1772996

**Published:** 2026-03-04

**Authors:** Dongmei Hu, Yingdian Yu

**Affiliations:** Department of Intensive Care, The Affiliated Traditional Chinese Medicine Hospital, Guangzhou Medical University, Guangzhou, China

**Keywords:** acute respiratory distress syndrome, case report, early rehabilitation, extracorporeal membrane oxygenation, single-site dual-lumen cannulation

## Abstract

This case report describes the successful management of a critically ill elderly patient with severe SARS-CoV-2-associated acute respiratory distress syndrome (ARDS) using an integrated strategy of awake veno-venous extracorporeal membrane oxygenation (VV-ECMO) via a single-site, dual-lumen cannula (SDLC) combined with a protocolized early rehabilitation programme. Despite high predicted mortality based on validated prognostic scores, the patient achieved a favorable outcome. The SDLC, inserted via the right internal jugular vein, was instrumental as a technological enabler. By providing unobstructed access to the lower limbs, it permitted unrestricted physiotherapy and early mobilization, which is vital for preserving musculoskeletal and neurocognitive function, supporting nutrition, and enhancing patient autonomy. Furthermore, a single puncture site reduces infectious risks and simplifies care compared to traditional multi-cannula configurations. However, this approach demands flawless technical execution, mandating real-time transesophageal echocardiography (TEE) guidance for precise cannula positioning to avoid recirculation or myocardial injury, and often requires intensified anticoagulation, increasing bleeding risk. The awake ECMO strategy deliberately harnesses spontaneous breathing to promote lung recruitment while facilitating ultra-protective ventilation, though it necessitates careful monitoring to mitigate the risk of patient self-inflicted lung injury (P-SILI). For carefully selected patients requiring prolonged support, the triad of SDLC VV-ECMO, an awake strategy, and early rehabilitation is a feasible and advantageous approach that shifts the treatment goal from mere survival to functional survival. It should be employed in expert centers, and future research is needed to standardize protocols and validate long-term outcomes.

## Introduction

The global pandemic of coronavirus disease 2019 (COVID-19) has resulted in significant global mortality, with a primary driver being the progression to severe acute respiratory distress syndrome (ARDS) in critically ill patients, presenting unprecedented challenges to intensive care systems worldwide (1). For a subset of these patients who develop profound, refractory hypoxemia despite maximal conventional management–including lung-protective ventilation and prone positioning–veno-venous extracorporeal membrane oxygenation (VV-ECMO) serves as an essential salvage therapy to support gas exchange and allow for lung recovery (2). However, the conventional paradigm for VV-ECMO management has historically been coupled with invasive mechanical ventilation under deep sedation and neuromuscular blockade (3). This approach, while life-saving, carries a substantial burden of iatrogenic complications. These include the development of intensive care unit-acquired weakness due to prolonged immobilization and myopathy, a high incidence of delirium, ventilator-associated pneumonia, and ultimately, a protracted recovery trajectory with significant functional impairment that impacts long-term quality of life (4).

In response to these well-documented drawbacks, the innovative concept of “awake ECMO” has been developed and increasingly adopted. This strategy deliberately avoids or minimizes the use of invasive ventilation and deep sedation (5). By preserving the patient’s spontaneous breathing effort, it maintains diaphragmatic and respiratory muscle function, reduces the need for vasopressors often required to counter sedation-induced hypotension, and, most importantly, enables the patient to actively participate in early rehabilitation protocols (6, 7). Early and aggressive physiotherapy has been specifically shown to counteract the rapid skeletal muscle catabolism inherent in critical illness and is crucial for mitigating functional decline in this vulnerable population (8).

To minimize the risks associated with mechanical ventilation, an awake ECMO strategy utilizing single-site cannulation has been proposed (9). However, the practical execution of an awake ECMO strategy is critically dependent on cannulation technique. The utilization of a SDLC, positioned meticulously under real-time transesophageal echocardiography (TEE) guidance, represents a key technological advancement (10). This configuration consolidates venous drainage and arterialized blood return through a single percutaneous access in the right internal jugular vein. This design offers distinct advantages: it minimizes vascular injury by reducing the number of cannulation sites, optimizes flow dynamics to decrease recirculation fraction, thereby improving oxygenation efficiency, and may lower the cumulative risk of catheter-related infections and bleeding complications compared to traditional multi-cannula femoral-jugular configurations (11).

Although international registries and case series have begun to report on the feasibility and potential benefits of awake ECMO, particularly in the context of SARS-CoV-2-induced ARDS, detailed clinical experience, standardized protocols, and outcome reports from within China remain relatively sparse. This article details the successful comprehensive management of an elderly patient with severe COVID-19-related ARDS. The management integrated TEE-guided awake VV-ECMO utilizing a SDLC with targeted antiviral and immunomodulatory pharmacotherapy. We present this case to illustrate the practical application, technical considerations, and multidisciplinary coordination required for this approach, aiming to support its broader and more standardized adoption in managing similar complex cases and to contribute valuable real-world evidence to the growing body of literature on advanced, patient-centered respiratory support strategies.

## Case description

**FIGURE 1:**
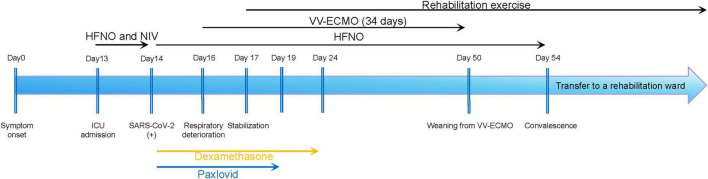
Clinical course timeline.

A 74-years-old man was transferred and admitted to the intensive care unit of Zhujiang Hospital of Southern Medical University, presenting with an 11-days history of persistent fever, productive cough, and a critical 2-days evolution of progressive dyspnea. His medical history was significant for multiple comorbidities, including early-stage non-small cell lung cancer after surgery, chronic kidney disease, essential hypertension, and type 2 diabetes mellitus, all of which compounded the complexity of his acute presentation. A schematic timeline of his clinical course is provided in Figure 1.

Upon admission, he was in severe respiratory distress, with an oxygen saturation of 82% despite the support of a high-flow nasal cannula (HFNC) set at 60 liters per minute with a fraction of inspired oxygen (FiO_2_) of 100%. Physical examination was notable for marked tachypnea (respiratory rate of 26 breaths per minute) and diffuse bilateral coarse crackles on auscultation. Initial arterial blood gas (ABG) analysis on HFNC revealed profound hypoxemic respiratory failure with a component of respiratory acidosis (pH 7.334, PaCO_2_ 31.9 mmHg, PaO_2_ 55 mmHg) and an elevated serum lactate level of 3.0 mmol/L, suggestive of tissue hypoperfusion. Laboratory investigations were indicative of a severe systemic inflammatory response, evidenced by leukocytosis (white blood cell count 24.49 × 10^9^/L), markedly elevated interleukin-6 (31.9 ng/L), and high-sensitivity C-reactive protein (38 mg/L). Additionally, they revealed acute-on-chronic renal failure (serum creatinine 282 μmol/L). A nasopharyngeal swab confirmed SARS-CoV-2 infection via polymerase chain reaction (PCR) testing. Chest radiography and subsequent computed tomography (CT) imaging demonstrated extensive, bilateral ground-glass opacities and consolidations involving all lung lobes, fulfilling the radiologic criteria for acute respiratory distress syndrome (ARDS). Point-of-care lung ultrasonography corroborated these findings, showing extensive pulmonary consolidation with prominent B-lines indicative of interstitial edema.

**FIGURE 2:**
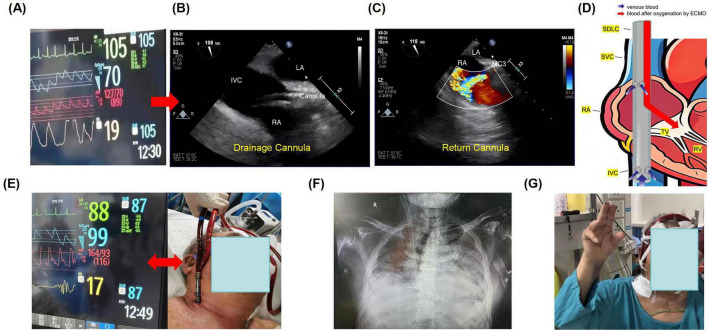
Case presentation: **(A)** vital signs during resuscitation; **(B)** placement of the drainage cannula under TEE; **(C)** placement of the return cannula under TEE; **(D)** schematic diagram of the SDLC catheter position; **(E)** vital signs after establishment of portable VV-ECMO support; **(F)** chest X-ray confirming cannula position; **(G)** early rehabilitation: sitting with portable VV-ECMO support.

Initial management adhered to established protocols for severe COVID-19 pneumonia, consisting of continued HFNC therapy, repeated awake prone positioning, dexamethasone (6 mg daily), the antiviral nirmatrelvir/ritonavir (Paxlovid^®^), empiric broad-spectrum antibiotics, and CRRT for fluid and uremic management. Despite this aggressive support, his respiratory status deteriorated rapidly. By hospital day 3, a repeat ABG on maximal HFNC (FiO_2_ 100%) revealed life-threatening hypercapnic and hypoxemic respiratory failure (pH 7.15, PaCO_2_ 82 mmHg, PaO_2_ 45 mmHg), with a PaO_2_/FiO_2_ ratio of less than 80. Vital signs at the time are presented in Figure 2A. The patient (who remained fully alert) and his family, after detailed discussion regarding the prognosis, explicitly declined endotracheal intubation and invasive mechanical ventilation. A focused bedside cardiac echocardiogram showed preserved biventricular systolic function without significant valvular pathology, confirming isolated severe respiratory failure.

Given the refractory hypoxemia and hypercapnia despite optimal medical management, and in alignment with the patient’s wish to avoid invasive ventilation, VV-ECMO utilizing a SDLC was evaluated as a potential rescue therapy. Formal prognostic scoring was performed: the ECMOnet Score was 6, the RESP Score was −2, and the PRESET Score was 7 (Supplementary Table), all indicating a high predicted mortality risk. Nevertheless, after a multidisciplinary team discussion involving intensivists, cardiothoracic surgeons, and the patient’s family, a joint decision was made to proceed with VV-ECMO as a bridge to lung recovery.

A 31-French dual-lumen Crescent^®^ ECMO catheter was percutaneously inserted into the right internal jugular vein under strict sterile conditions. Real-time transesophageal echocardiography (TEE) was employed throughout to advance the guidewire into the inferior vena cava and to meticulously position the drainage cannula (Figure 2B), ensuring the return cannula was directed anteroinferiorly toward the tricuspid valve to reduce recirculation (Figure 2C). The schematic diagram of catheter placement is shown in Figure 2D. Portable VV-ECMO support was successfully established within 20 min, with immediate improvement in the patient’s vital signs (Figure 2E). A bedside chest X-ray confirmed proper and stable catheter position (Figure 2F). The initial configuration was set as follows: blood flow, 3.5 L/min; rotational speed, 3000 rpm; FiO_2_, 100%; sweep gas flow, 5 L/min; and water tank temperature, 37°C. These parameters were adjusted according to serial arterial blood gas (ABG) results and the patient’s clinical status. Concurrent lung-protective spontaneous breathing was supported with low-level pressure support via HFNC therapy. Sedation was minimized using a combination of low-dose butorphanol for analgesia and dexmedetomidine for light anxiolysis. This approach maintained the patient in an awake, cooperative, and interactive state–the cornerstone of the “awake ECMO” strategy.

**FIGURE 3:**
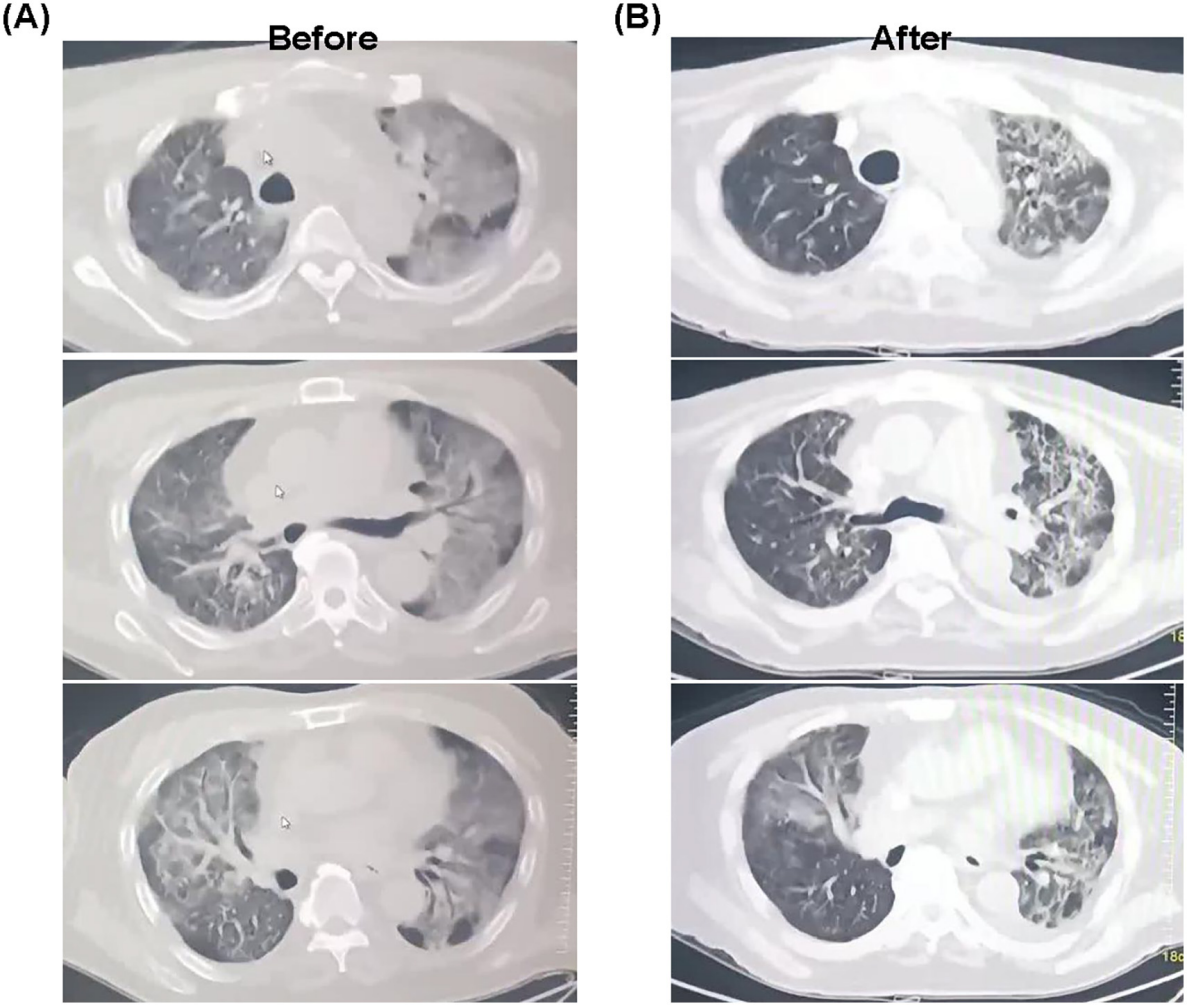
**(A)** Radioactive imaging examination before rescue. **(B)** Radioactive imaging examination after rescue.

Following cannulation, the patient’s work of breathing decreased dramatically, with substantial improvement in oxygenation. A protocolized early rehabilitation program was initiated within 24 h. With the advantage of a single-site, dual-lumen cannula that left both groins free, the patient engaged in active limb exercises in bed daily. His mobility progressed steadily: from sitting at the edge of the bed, to standing with support, and eventually to sitting in a chair (Figure 2G). Throughout his 34-days ECMO course, he remained alert, communicated effectively, and participated actively in his care. Oral nutritional intake was well-maintained with dietitian support. In addition, chest CT performed prior to ECMO cannulation revealed extensive bilateral ground-glass opacities and multifocal consolidations, with areas of confluence leading to markedly reduced lung transparency. These findings were consistent with the pattern of diffuse alveolar damage characteristic of severe ARDS, reflecting profound alveolar and interstitial edema, inflammation, and parenchymal injury. Follow-up CT after a period of ECMO support demonstrated significant radiological improvement. There was a notable increase in overall lung lucency, along with a substantial reduction in the extent and density of the previous ground-glass and consolidative opacities. Consequently, a considerable volume of normally aerated lung parenchyma was re-established (Figure 3). After meeting standardized weaning criteria–including adequate native lung function on minimal ECMO support (flows < 2 L/min)–the patient was successfully decannulated on ECMO day 34. He was transferred from the ICU to a step-down unit on hospital day 41, ambulating with minimal supplemental oxygen. At the last follow-up in November 2025, the patient had experienced no relapse of respiratory failure and continues to participate in rehabilitation with steadily improving functional status.

## Discussion

This case report details the successful management of a critically ill elderly patient with severe SARS-CoV-2-associated ARDS, utilizing an integrated strategy of awake VV-ECMO via a single-site dual-lumen cannula (SDLC) and a protocolized early rehabilitation program. The patient’s favorable clinical outcome, achieved despite high predicted mortality based on validated prognostic scores, calls into question a purely score-driven approach. It underscores the imperative for nuanced, patient-centered decision-making, in which the patient’s functional potential is carefully balanced against statistical risks. The following discussion elucidates the role of the SDLC as a key technological enabler, analyzes the principles and benefits of the awake ECMO strategy, and outlines critical technical considerations for its implementation.

Prognostic scores such as ECMOnet, RESP, and PRESET are invaluable for population-level risk stratification (12). However, applying these scores to guide decisions for individual patients within novel management frameworks requires careful interpretation. This patient’s high-risk profile was defined by factors traditionally associated with poor outcomes under conventional ECMO management with deep sedation and paralysis. The favorable outcome in this case suggests that the negative prognostic weight of such factors can be mitigated by a strategy designed to proactively prevent iatrogenic harm. The awake SDLC-ECMO approach alters the clinical trajectory by avoiding prolonged intubation and deep sedation, thereby directly mitigating primary drivers of ICU-acquired weakness and delirium (13). Consequently, this case advocates for integrating assessments of a patient’s functional and therapeutic potential into ECMO candidacy evaluations. It suggests that the capacity to participate in an awake strategy may itself be a positive prognostic indicator.

It is worth noting that cannulation strategy plays a decisive role. Traditional two-site VV-ECMO imposes severe constraints on mobility and comfort due to the femoral cannula. In contrast, the SDLC, inserted via the right internal jugular vein, provides a transformative advantage: it liberates the lower limbs, enabling full mobility. This single modification unlocks comprehensive rehabilitation by permitting unrestricted physiotherapy, which is vital for preserving musculoskeletal and neurocognitive function. Upright posture sustains cardiopulmonary interaction (14) and gastrointestinal motility, supporting nutrition and gut barrier integrity (15). A single puncture site halves potential entry points for catheter-related bloodstream infection, eliminates femoral complications, and simplifies care. Enhanced comfort and autonomy foster therapeutic compliance and reduce psychological morbidity (16). A retrospective analysis of 2,628 patients found that patients managed with portable ECMO were more likely to be extubated within 24 h than those receiving conventional VV-ECMO (17).

However, SDLC placement is technically complex and mandates real-time TEE guidance for confirming correct guidewire passage and, crucially, ensuring the distal end of the catheter is inserted into the proximal end of the inferior vena cava, and precise orientation of the return jet toward the tricuspid valve. Malposition risks recirculation or myocardial erosion. Furthermore, the unique hemodynamic profile of the SDLC may increase thrombogenicity, often necessitating more intense anticoagulation, which in turn elevates the bleeding risk (10). Fortunately, no symptoms of bleeding or thrombosis occurred during the treatment of this patient.

The “awake ECMO” strategy represents a deliberate paradigm shift toward physiological preservation. It seeks to harness spontaneous breathing to promote lung recruitment and prevent diaphragmatic atrophy, while facilitating ultra-protective lung ventilation. The principal concern is P-SILI. In this case, stable ventilatory parameters suggested that ECMO adequately modulated the respiratory drive to mitigate P-SILI risk, a delicate balance that requires vigilant monitoring. The preserved cough reflex aids pulmonary hygiene, and “silent hypoxemia” may have aided psychological tolerance. Within this model, rehabilitation transcends supportive care to become a primary therapeutic intervention. The SDLC enables this, but success hinges on a structured, multidisciplinary protocol. Early mobilization counteracts catabolism and is linked to improved outcomes (9). This embodies a paradigm shift, viewing the patient as an active participant in recovery rather than a passive recipient of care. This approach avoids the cascade of complications associated with conventional management, with potential benefits extending to long-term functional recovery and quality of life.

In conclusion, for a carefully selected subset of patients with severe but reversible respiratory failure, especially those requiring prolonged support, the combined approach of SDLC VV-ECMO, an awake strategy, and aggressive early rehabilitation is feasible and advantageous. It shifts the therapeutic goal from mere survival to functional recovery. However, this approach demands exceptional institutional expertise. Future research must prospectively define optimal patient selection criteria, establish standardized protocols, and validate whether short-term benefits translate into improved long-term outcomes. Ultimately, this case reinforces that technological innovation must be guided by a holistic, patient-centered philosophy to achieve optimal results.

## Data Availability

The raw data supporting the conclusions of this article will be made available by the authors, without undue reservation.
