# Supplementary material for: Extracorporeal membrane oxygenation with single-site dual-lumen in a patient with SARS-CoV-2–associated acute respiratory distress syndrome: a case report
Source: Front Med (Lausanne). 2026 Mar 4;13:1772996. doi: 10.3389/fmed.2026.1772996 (PMC12996247; doi:10.3389/fmed.2026.1772996)
Supplement: Supplementary file 1 [file Data_Sheet_1.pdf]

Supplement Table

ECMOnet Score			
**Variable**	**Options**	**Score**	**Total score**
Dias hospitalizacion(day)	＜3 (0.5)	0.5	6
	4-7 (1)		
	8-11 (1.5)		
	＞11 (1)		
Bilirrubina (mg/dl)	≤2.57 (0)	0.5	
	2.58-11 (0.5)		
	11-19 (1)		
	19-28 (1.5)		
	28-37 (2)		
	＞37 (2.5)		
Creatinina (mg/dl)	≤44 (0)	3.5	
	44-71 (0.5)		
	71-97 (1)		
	97-124 (1.5)		
	124-150 (2)		
	150-177 (2.5)		
	177-203 (3)		
	＞203 (3.5)		
Hematocrito (%)	＞40% (0.5)	1.5	
	36-40% (1)		
	31-35% (1.5)		
	≤30% (2)		
PAM (mmHg)	＞90 (0)	0	
	61-90 (0.5)		
	≤60 (1)		
**RESP Score**			
**Variable**	**Options**	**Score**	**Total score**
Age (Year)	19-49 (0)	-3	-2
	50-59 (-2)		
	＞60 (-3)		
PaCO2 (mmHg)	＜75 (0)	0	
	＞75 (-1)		
Peak Inspiratory Pressure (cmH2O)	＜45 (0)	-	
	＞45 (-1)		
Immunocompromised	Yes (-2)	-2	
	No (0)		
CNS dysfunction	Yes (-7)	-	
	No (0)		
Acute nonpulmonary infection	Yes (-3)	-	
	No (0)		
Neuromuscular block used before ECMO	Yes (1)	-	
	No (0)		
Nitric oxide use before ECMO	Yes (-1)	-	

Supplement Table

	No (0)		
Bicarbonate infusion before ECMO	Yes (-2)	-	
	No (0)		
Cardiac arrest before ECMO	Yes (-2)	-	
	No (0)		
On Ventilator Prior To ECMO	< 48 hours (3)	-	
	48 hours to 7 days (1)		
	>7 days (0)		
Acute Pulmonary Disease	Viral or bacterial pneumonia (3)	3	
	Asthma (1)		
	Trauma/burn (3)		
	Aspiration pneumonia (5)		
	Other acute pulmonary disease (1)		
	Chronic pulmonary or nonpulmonary disease (0)		

PRESET Score

Variable	Options	Score	Total score
PAM (mmHg)	＞100 (0)	2	7
	91-100 (1)		
	81-90 (2)		
	71-80 (3)		
	≤70 (4)		
Lactato (mmol/L)	≤1.5 (0)	2	
	1.5-3 (1)		
	3.01-6 (2)		
	6.01-10 (3)		
	≥10 (4)		
PH	＞7.30 (0)	2	
	7.201-7.300 (1)		
	7.101-7.200 (2)		
	≤7.1 (3)		
Plaquetas×1000uL(-1)	＞200 (0)	0	
	101-200 (1)		
	≤100 (2)		
Dias hospitalizacion PreECMO(day)	＜2 (0)	1	
	3-7 (1)		
	＞7 (2)		
